# Intron 4 Containing Novel GABAB1 Isoforms Impair GABAB Receptor Function

**DOI:** 10.1371/journal.pone.0014044

**Published:** 2010-11-18

**Authors:** Changhoon Lee, R. Dayne Mayfield, R. Adron Harris

**Affiliations:** Section of Neurobiology and Institute for Cellular and Molecular Biology, Waggoner Center for Alcohol and Addiction Research, The University of Texas at Austin, Austin, Texas, United States of America; University of Victoria, Canada

## Abstract

**Background:**

Gamma-aminobutyric acid type B (GABAB) receptors decrease neural activity through G protein signaling. There are two subunits, GABAB1 and GABAB2. Alternative splicing provides GABAB1 with structural and functional diversity. cDNA microarrays showed strong signals from human brain RNA using GABAB1 intron 4 region probes. Therefore, we predicted the existence of novel splice variants.

**Methodology/Principal Findings:**

Based on the probe sequence analysis, we proposed two possible splice variants, GABAB1j and GABAB1k. The existence of human GABAB1j was verified by quantitative real-time PCR, and mouse GABAB1j was found from a microarray probe set based on human GABAB1j sequence. GABAB1j open reading frames (ORF) and expression patterns are not conserved across species, and they do not have any important functional domains except sushi domains. Thus, we focused on another possible splice variant, GABAB1k. After obtaining PCR evidence for GABAB1k existence from human, mouse, and rat, it was cloned from human and mouse by PCR along with three additional isoforms, GABAB1l, GABAB1m, and GABAB1n. Their expression levels by quantitative real-time PCR are relatively low in brain although they may be expressed in specific cell types. GABAB1l and GABAB1m inhibit GABAB receptor-induced G protein-activated inwardly rectifying K^+^ channel (GIRK) currents at *Xenopus* oocyte two-electrode voltage clamp system.

**Conclusions/Significance:**

This study supports previous suggestions that intron 4 of GABAB1 gene is a frequent splicing spot across species. Like GABAB1e, GABAB1l and GABAB1m do not have transmembrane domains but have a dimerization motif. So, they also could be secreted and bind GABAB2 dominantly instead of GABAB1a. However, only GABAB1l and GABAB1m are N- and C-terminal truncated splicing variants and impair receptor function. This suggests that the intron 4 containing N-terminal truncation is necessary for the inhibitory action of the new splice variants.

## Introduction

The GABAB receptor is a metabotropic receptor that is highly expressed in brain and weakly expressed in heart, small intestine, uterus and other tissues [1]. The functional receptor is a hetero-oligomer of GABAB1 and GABAB2 subunits, where the intracellular domain of GABAB1 dimerizes with GABAB2. GABA binds to the extracellular domain of GABAB1 and transfers signals through G proteins. G protein α subunits are linked with adenylyl cyclase (AC), and G protein βγ subunits alter Ca^2+^ channels and GIRK channels. The GABAB receptor decreases the activity of AC and decreases neurotransmitter release by inhibiting Ca^2+^ influx through presynaptic Ca^2+^ channels. At postsynaptic neurons, it activates K^+^ channels, and an outward K^+^ current induces hyperpolarization preventing Na^+^ channel opening and action potential firing [2]. Thus, the GABAB receptor mediates an inhibitory neurotransmission.

**Figure 1:**
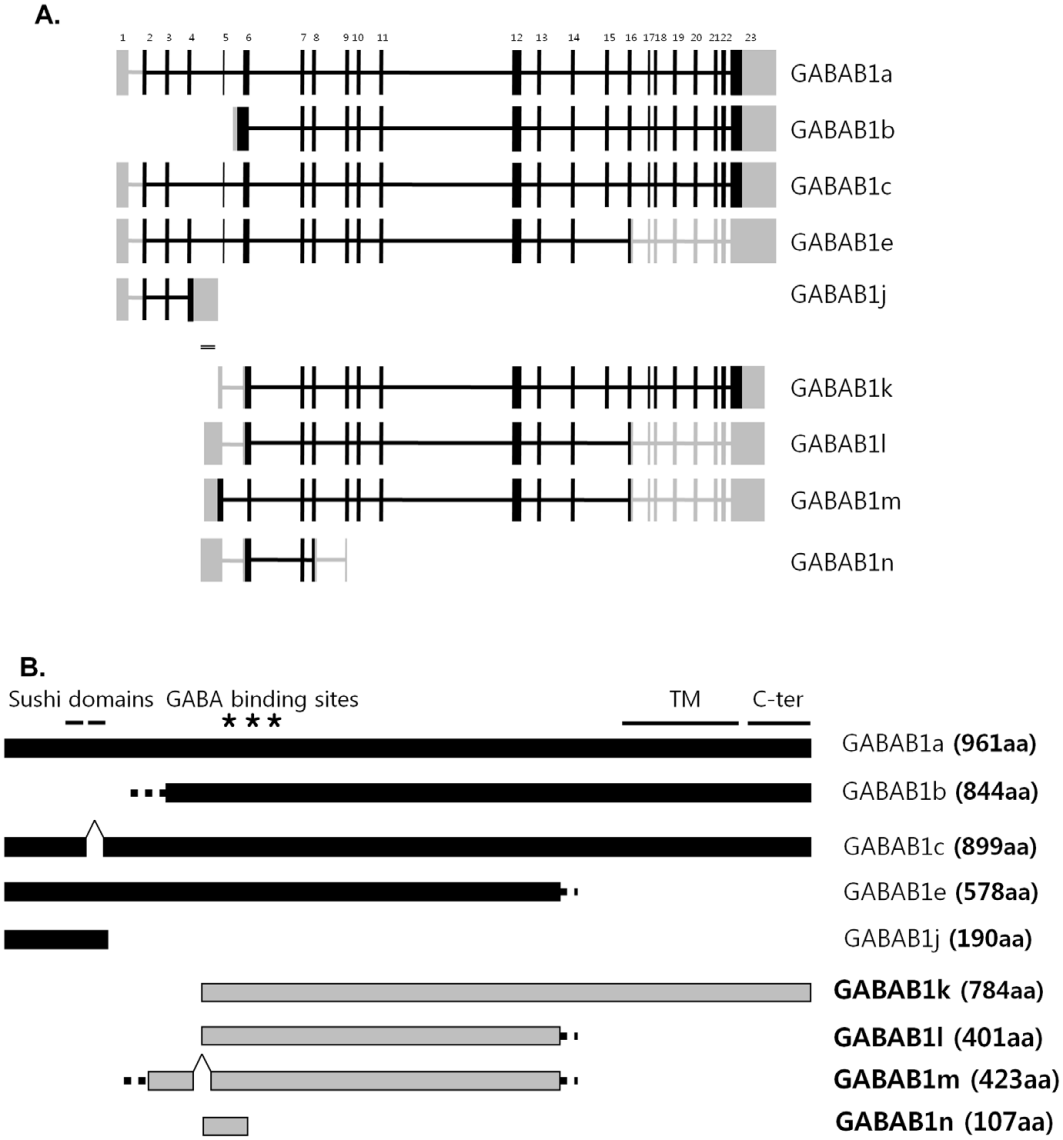
Schematic summary of GABAB1 isoforms. A. Introns and exons of known and novel GABAB1 isoforms were compared. The numbers listed at the top are exon numbers of GABAB1a. Blocks represent exons, and lines are introns of the GABAB1 gene. Black lines and blocks are ORFs, and grey lines and blocks are UTRs. Double line is cDNA microarray probe, clone 300899, which aligns to the intron 4 of GABAB1a. Human GABAB1a, b, c, e, and rat GABAB1j are previously identified isoforms. After cloning, the following novel isoforms were found in human: GABAB1k, GABAB1l, GABAB1m, and GABAB1n. Human GABAB1j was predicted. Mouse GABAB1j and GABAB1k were also cloned. GABAB1k contains the 3′ part of intron 4 through exon 23. GABAB1l has additional splicing out of exon 15, and GABAB1m has similar splicing patterns as GABAB1l except for additional splicing out at 5′ part of exon 6. GABAB1n has an SNP on exon 8. B. ORFs of GABAB1 isoforms are shown with important functional domains. Black bars are previously known isoforms, and grey bars represent novel isoforms. Dotted lines stand for unique sequence of each isoform. GABAB1k contains GABA binding sites, seven transmembrane domains, a G-protein coupling region, and a C-terminal intracellular region. GABAB1l and GABAB1m do not have transmembrane domains, a G-protein coupling region, or C-terminal intracellular regions (same as GABAB1e). GABAB1m has a longer N-terminal than GABAB1l. GABAB1n contains only a partial GABA binding site.

GABAB1 has two major splicing variants, GABAB1a and GABAB1b, in the human central nervous system. GABAB1a is the longest form and has 23 exons (Figure 1A). GABAB1b is an N-terminal truncated form of GABAB1a and has an alternative exon from intron 5 resulting in a unique N-terminal without sushi domains (Figure 1A, B). GABAB1a and b are the most widely studied isoforms to date and show some distinct expression patterns in brain as GABAB1a is more presynaptic in localization, and GABAB1b is more postsynaptic [3], [4], [5]. However, the differences in their localization and function are still not known precisely [4]. Additional splice variants of GABAB1 have been reported although some were found only in one species, and most do not have defined functions [3], [6], [7]. Human GABAB1c lacks exon 4 (Figure 1A), and its functions have not been studied in detail [4]. Human GABAB1e is mainly expressed in peripheral tissues. Because it lacks exon15, GABAB1e is a C-terminal truncated form of GABAB1a and does not have seven transmembrane domains, a G-protein coupling region, nor a C-terminal intracellular region (Figure 1A, B). However, it is functionally active and prevents GABAB1a and GABAB2 heterodimerization [8]. In rat, the GABAB1j isoform was recently discovered and found to be expressed as highly as GABAB1a and b in brain. It contains exon 1 through 4 and the 5′ part of intron 4 and is thus a small C-terminal truncated GABAB1 isoform (Figure 1A). The GABAB1j is a soluble isoform with sushi domains (Figure 1B), which selectively impair the function of presynaptic (but not postsynaptic) GABAB receptors [7]. Thus, alternative splicing provides a large diversity of structural and functional variation in GABAB1 receptors.

The diversity of GABAB1 receptors is indicated by these previous studies, and it is possible that additional unknown isoforms exist. Two previous cDNA microarray studies found strong GABAB1 signals in human brain mRNA [9], [10]. The two probe sequences used in these studies, NCBI accession numbers N80593 and W07715, were obtained from one single probe with clone ID 300899. The probe aligns to intron 4 of GABAB1a. This is a common intron region of the major human GABAB1 splice variants (Figure 1A) and has been noted as a potentially important location for alternative splicing in rat [6]. Because the probe was made from a cDNA library, the probe region should be an alternative exon in the GABAB1 gene suggesting the existence of novel GABAB1 isoforms. The goals of this study were to identify the novel isoforms and determine their functions.

## Results

### Microarray probe sequence analysis

The expression of clone 300899 increased significantly in human prefrontal cortices in two independent microarray studies [9], [10], and the clone aligned to the intron 4 region (Figure 1A). Sequencing of clone 300899 showed multiple stop codons from all six translated frames and likely represented untranslated region (UTR)s. Thus, two possible isoform models, GABAB1j and GABAB1k, were proposed for human allowing us to study novel GABAB1 splicing variant isoforms. Human GABAB1j and GABAB1k contain clone 300899 at their 3′ and 5′ UTR, respectively. In human brain, GABAB1k was expected to have intron 4 and exon 5 through 23, and GABAB1j was also proposed to contain exon 1 through 4 and intron 4 like rat GABAB1j.

### GABAB1j: Cloning and mRNA levels in human and mouse brain

**Figure 2:**
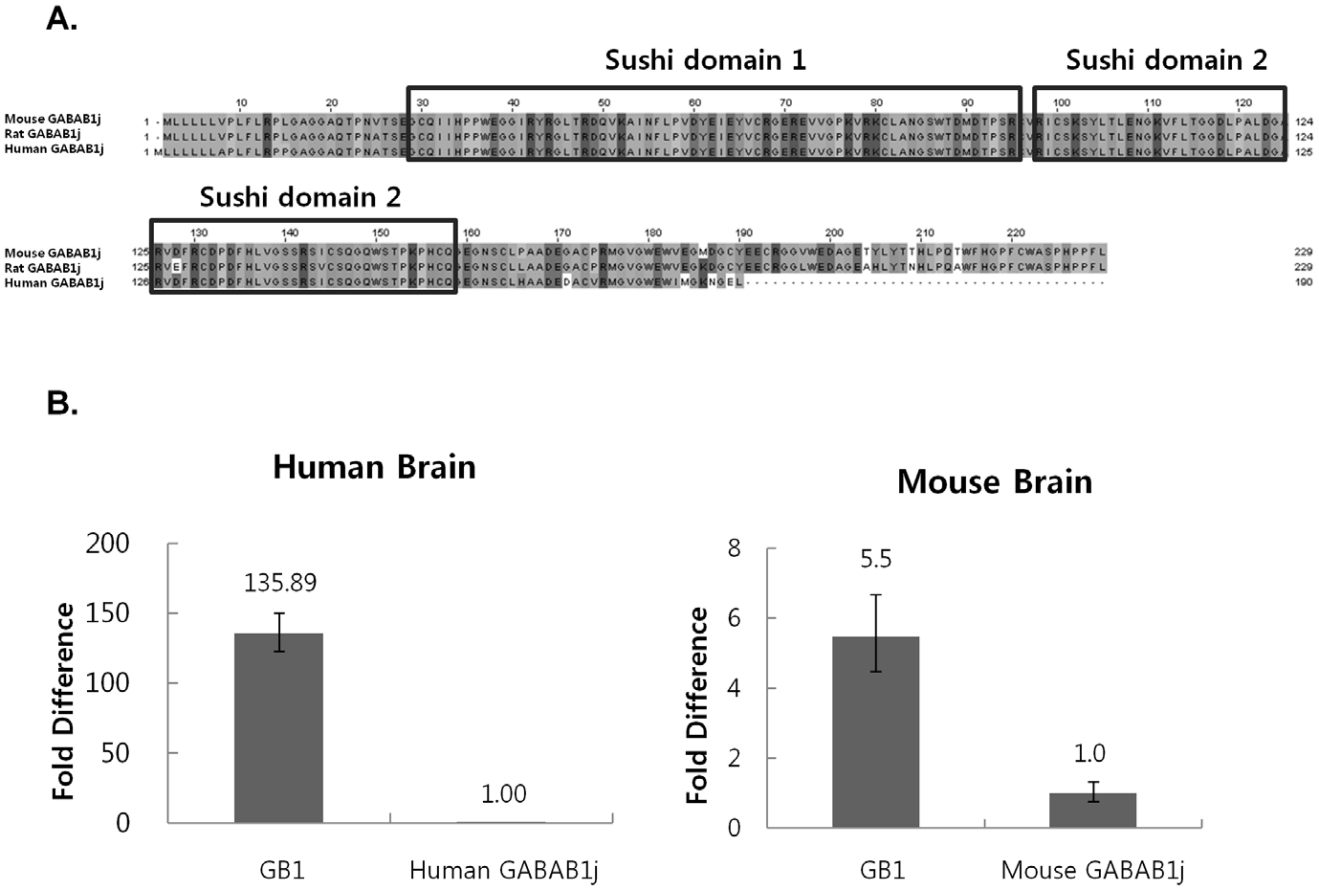
Sequences for human, mouse, and rat GABAB1j isoforms and relative expression levels. A. Sequence alignment of human, mouse, and rat GABAB1j showed a different C-terminal pattern in human GABAB1j. B. Human and mouse GABAB1j mRNA expression levels were measured with quantitative real-time PCR. GB1 primer and probe sets were used to detect most known major GABAB1 isoforms except GABAB1j. Human and mouse GABAB1j primer and probe sets were specific for only GABAB1j. GB1 expressions were shown as fold differences from human and mouse GABAB1j. Although a previous rat GABAB1j study indicated that GB1 and GABAB1j expression levels were similar, human and mouse GABAB1j expressions were lower than GB1. Mouse GABAB1j expression was 5.5 fold lower than GB1 expression, and human GABAB1j showed 135.89 fold lower expression. If the fold differences were compared, human GABAB1j expression level was much less (25 times) than mouse GABAB1j expression. Therefore, GABAB1j expressions vary across species.

GABAB1j was previously cloned in rat, but not in human or mouse. Rat GABAB1j ORF is 229 amino acids (aa) (Figure 2A) [7]. Though the predicted human GABAB1j has some different aa sequences and a shorter ORF of 190 aa, it shares similar motifs with rat GABAB1j such as C-terminal truncated splicing and lack of important functional domains of common GABAB1 isoforms. Although human GABAB1a has 23 exons, it has only the first 4 exons and partial 5′ intron 4 (Figure 1A). Two novel clones were found from the mouse cDNA microarray probe set after comparing sequences with the possible human GABAB1j [11]. The lengths of the two clones are different because their UTR lengths are not the same, but they shared identical ORF. They have the same exon composition and ORF length as rat GABAB1j. Sequencing alignment also showed that mouse GABAB1j shares the similar ORF except a few aa differences (Figure 2A).

The intron 4 sequence of the predicted human GABAB1j is different from mouse and rat GABAB1j. Therefore, sequence alignment showed that it has a very unique C-terminal and a 39 aa shorter ORF than mouse and rat GABAB1j (Figure 2A). To do quantitative real-time PCRs, primer and probe sets for general GABAB1 isoform (GB1) were designed between the last two exons of GABAB1a, exon 22 and 23, and they were specific for most known GABAB1 isoforms except GABAB1j. GABAB1j specific primer and probe sets were also prepared. Using quantitative real-time PCR, the existence of the predicted human GABAB1j was verified. Relative mouse GABAB1j mRNA expression level was much higher than human GABAB1j when compared to GB1 expression (Figure 2B). GB1 sets and the northern blot probe of the rat GABAB1j paper [7] were supposed to detect the same major known isoforms. Though rat GABAB1j expression level in brain were as high as GB1, human and mouse GABAB1j expression were less than GB1 expression (Figure 2B).

GABAB1j function in human and rodent might not be identical because of their expression and sequence differences. Rat GABAB1j expression is as strong as major GABAB1 isoforms [7]. Though mouse GABAB1j expression is only 5.5 fold lower than them (Figure 2B), human GABAB1j showed much lower expression (136 fold) (Figure 2C). If the fold differences were compared, the relative human GABAB1j expression level to GB1 was also dramatically less (25 times) than mouse GABAB1j expression though identical detection regions were used for both expression studies. Thus, GABAB1j expressions are not consistent across species. Interestingly, the C-terminal sequence of human GABAB1j did not share a common pattern with mouse and rat GABAB1j (Figure 2A). From previous research, purified sushi domains of rat GABAB1j impaired the inhibitory effect of the GABAB receptor, but the function of rat GABAB1j full ORF had not been studied in detail [7]. So, it is not clear whether GABAB1j functions could be conserved across species.

### GABAB1k sequence analysis in human, mouse, and rat

The predicted human GABAB1k is an N-terminal truncated splicing variant. It contains 3′ part of intron 4 and exons 5 through 23, and the ORF was 784 aa (Figure 1A). It had important functional domains, such as GABA binding sites (Ser70, Tyr190, and Asp295), seven transmembrane domains (415–677 aa), a G-protein coupling region (linker region between transmembrane domain 3 and 4), and a C-terminal intracellular region (678–784 aa: dimerize with GABAB2) (Figure 1B). It shared most domains with GABAB1b, a functional receptor subunit that had no the sushi domains [12] (Figure 1B). Thus, GABAB1k might also be a functional receptor subunit with greater physiological effect than human GABAB1j, given that GABAB1j only contains the sushi domains (without any important domains of GABAB1b) and exhibits much lower relative expression in human than in mouse and rat. Therefore, we decided to focus on the longer splice variant GABAB1k instead of the very short splice variant GABAB1j.

**Figure 3:**
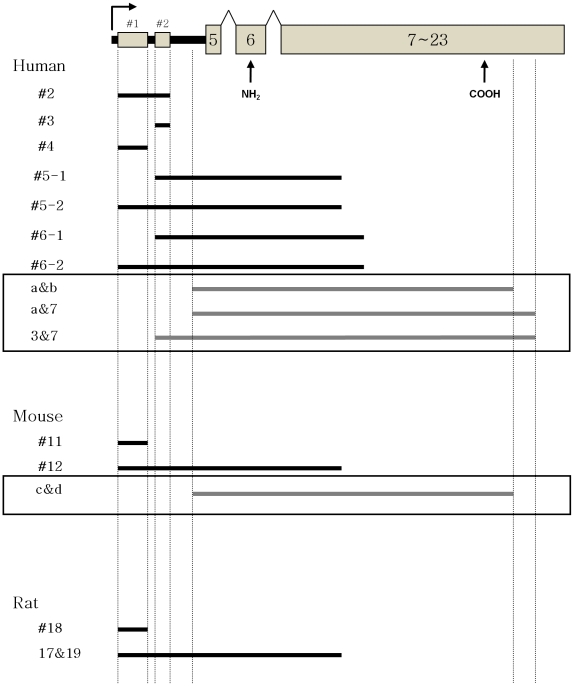
Strategy for cloning GABAB1k. The top figure represents exon-intron composition of the proposed GABAB1k. Numbered rectangular boxes represent known exons, and lines between boxes are introns. Two arrows on the bottom of the boxes represent ORF. Black bar is intron 4, and the two probe sequence data, NCBI accession numbers W07715 and N80593, are indicated as #1 and #2 above small rectangular boxes, respectively. They are 5′ and 3′ sequences of the microarray probe, clone 300899. A arrow above the black bar is a possible transcription initiation site. Horizontal lines indicate sequences detected after PCR. Based on microarray probe, clone 300899, the following clones were detected in human cultured cells, mouse midbrain, and rat hippocampus: #2, #3, #4, #11, and #18. From the previous microarray probe sequence analysis, the microarray probe aligned to intron 4 and was either 3′ or 5′ UTR of novel isoforms. Because there was no known human GABAB1 isoform that had intron 4 as an exon, the two possible isoform models, GABAB1j and GABAB1k, were proposed. The predicted GABAB1j and GAGAG1k contain intron 4 as 3′ or 5′ UTR, respectively. Because GABAB1j and GABAB1k are only two possible isoform models that contain clone 300899, #2, #3, #4, #11, and #18 can be partial GABAB1j or GABAB1k and show their existences. From the same RNA sources six different N-terminal partial clones, #5-1, #5-2, #6-1, #6-2, #12, and 17&19, indicated the existence of GABAB1k. They share common 5′ UTRs and partial N-terminal ORFs of GABAB1k and suggest that GABAB1k exists in human, mouse, and rat. To clone the full ORF of GABAB1k, additional primers were designed at its possible 3′ UTR region based on other known isoform sequence analyses. Full ORF containing clones were cloned from human brain and mouse midbrain. Bottom two boxes show clones which contain the full ORF (a&b, a&7, 3&7 in human and c&d in mouse).

Before cloning the predicted GABAB1k, its existence was verified in human, mouse, and rat. First, mRNAs containing microarray probe region, clone 300899, were detected across species. After purifying total RNA from human cultured cell lines, mouse midbrain, and rat hippocampus, DNase treatment removed possible genomic DNA contamination. After making mRNA specific cDNA with oligo(dT) primer, PCRs were performed within the probe regions. Three different clones (#2, #3, and #4) were isolated from human (Figure 3). After probe homologous regions in mouse and rat were found with Blast N Search, #11 and #18 were cloned in mouse and rat, respectively (Figure 3). The previous microarray probe sequence analysis proved that the microarray probe was a part of intron 4 and either 3′ or 5′ UTR of novel isoforms. Because there was no known human GABAB1 isoform that had the intron 4 region as an exon, the two possible clones, GABAB1j and GABAB1k, were predicted to contain the intron 4 regions as 3′ or 5′ UTR, respectively. GABAB1j and GABAB1k were also only possible isoform models that contain the microarray probe in intron 4. Thus, #2, #3, #4, #11, and #18 can be partial GABAB1j or GABAB1k, and mRNAs containing the probe regions exist in human, mouse, and rat.

To confirm the predicted GABAB1k existence and to remove the possibility of genomic DNA contamination, PCRs were performed between the probe region and other exons. Partial GABAB1k forms containing exon-intron junctions were also cloned from human cultured cell lines, mouse midbrain, and rat hippocampus. There were six different N-terminal partial clones, #5-1, #5-2, #6-1, #6-2, #12, and 17&19 (Figure 3). They share the partial ORF sequences of other known isoforms in the N-terminal and also have common 5′ UTRs. There is no possibility of genomic DNA contamination because introns are spliced out. Thus, the N-terminal partial clones suggested that GABAB1k exists. They are not long enough to have other functional domains, such as transmembrane domains, a G-protein coupling region, and a C-terminal intracellular region. It was, thus, necessary to obtain the full ORF for functional study of GABAB1k.

### Cloning GABAB1k, GABAB1l, GABAB1m, and GABAB1n

To clone the full ORF, additional primers were designed at the possible 3′ UTR of the predicted GABAB1k based on known isoform sequence analysis. From human whole brain and mouse midbrain, several clones were detected by PCR. They were a&b, a&7, 3&7, and c&d (Figure 3). The expected human GABAB1k and mouse GABAB1k were cloned. They have the 3′ part of intron 4 through exon 23 (Figure 1A). They are an N-terminal truncated splicing variant isoforms, and their ORFs are 784 aa. They contain functional domains, such as GABA binding sites, seven transmembrane domains, a G-protein coupling region, and a C-terminal intracellular region (Figure 1B).

In addition to GABAB1k, two other forms, GABAB1l and m, were also cloned in human. GABAB1l has additional splicing out of exon 15 and is both N-terminal and C-terminal truncated (Figure 1A, B). The C-terminal truncated pattern is the same as GABAB1e [8], and its ORF is 401 aa. Though it contains GABA binding sites, it does not have the other functional domains (Figure 1B). GABAB1m has almost the same splicing pattern as GABAB1l except for an additional missing at 5′ part of exon 6 (Figure 1A). Thus, its ORF is longer (423 aa) than human GABAB1l and starts at intron 4 whereas the ORFs of GABAB1k begin at exon 6 (Figure 1B).

During cloning, one more isoform, GABAB1n, was also found. It has a single nucleotide polymorphism (SNP) at exon 8 and so has a very small ORF, 107 aa. It does not have any important functional domains except a partial GABA binding site (Ser70) (Figure 1A, B).

### GABAB1k, GABAB1l, GABAB1m, and GABAB1n mRNA levels in human and mouse brains

After cloning the novel GABAB1 forms, GABAB1k, GABAB1l, GABAB1m, and GABAB1n, their mRNA expression levels were compared with other known isoforms in brain.

**Figure 4:**
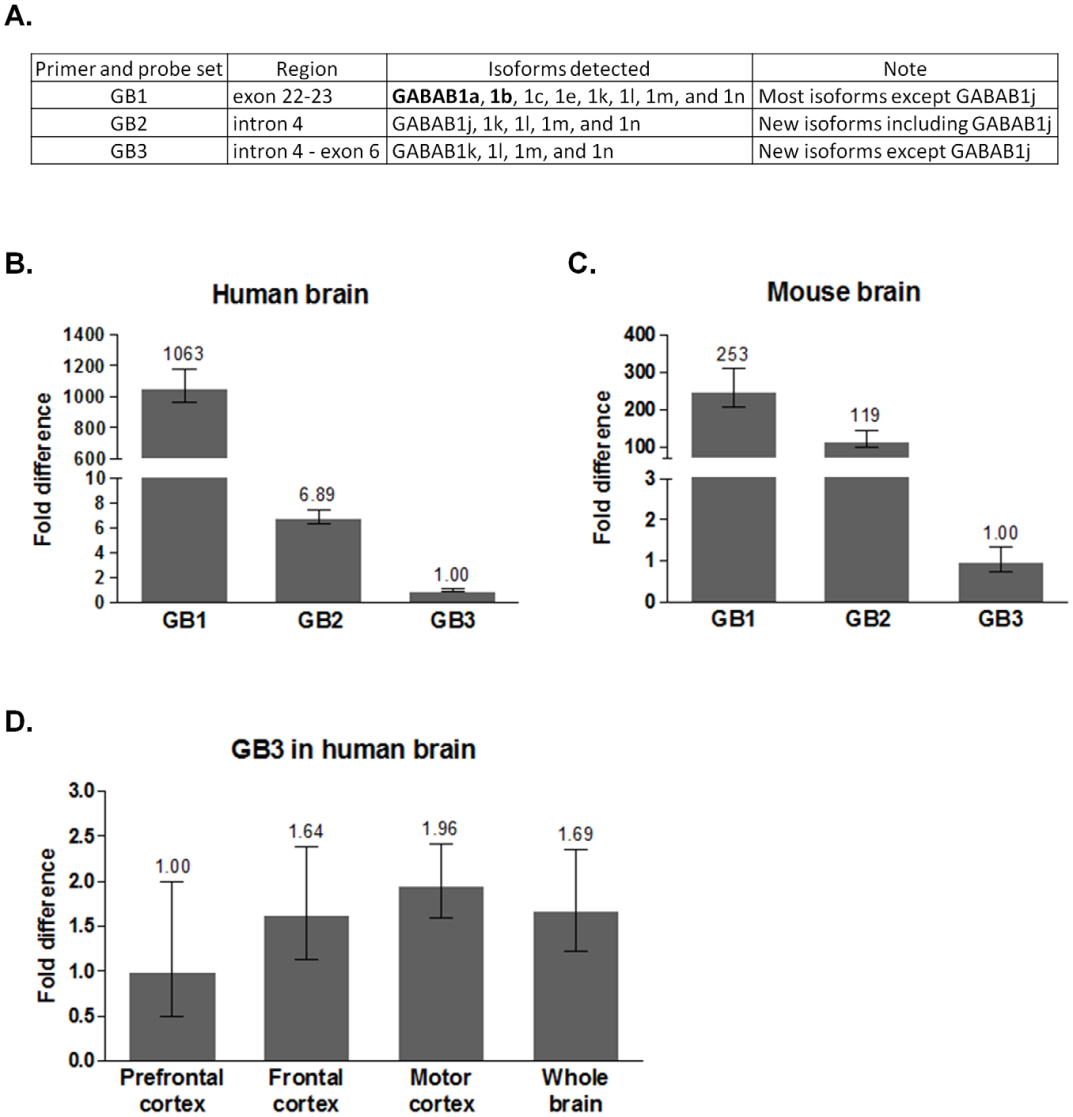
The expression of GABAB1 isoforms. A. For quantitative real-time PCR, primer and probe sets were designed from human and mouse. GB1 sets were designed by the last two exons, exon 22 and 23, and were used for detecting most known GABAB1 isoforms except GABAB1j. They also represented the microarray probes that aligned to 5′ exons of GABAB1 gene. GB2 sets were for detection of GABAB1j, k, l, m, and n. They aligned to intron 4 and designed for human cDNA microarray probe, clone 300899, and its mouse homologous probes. GB3 sets targeted intron 4 through exon 6 and were specific for GABAB1k, l, m, and n. B. In human brain, GB1 showed much more dominant expression than GB2 and GB3. C. GB1 expression was also stronger than GB2 and GB3 in mouse brain. However, GB2 and GB3 expression levels in mouse brain were much higher than human brain if they were compared with GB1 expressions. D. In human, GB3 expressions were similar in prefrontal cortex, frontal cortex, and motor cortex.

Previous human cDNA microarrays had three GABAB1 probes, clones 2312175, 298231, and 300899 [9], [10]. Clones 2312175 and 298231 aligned to exon 23, and clone 300899 aligned to intron 4. Our mouse cDNA microarray probe set had six different GABAB1 probes [11]. Three probes aligned to 3′ exons of the GABAB1 gene including exon 23, and the other three probes contained intron 4. Therefore, the human and mouse probe sets are two types that include exon 23 or intron 4 of GABAB1 gene. Based on the probe sequence information, GB1 primer and probe sets of the previous GABAB1j expression measurement experiments were used for quantitative real-time PCR. GB2 and GB3 primer and probe sets were designed (Figure 4A). GB1 sets targeting exon 22 and 23 junction represented human and mouse cDNA microarray probes that aligned to 3′ exons of GABAB1 gene. They detect all known GABAB1 isoforms except GABAB1j. Since they identify the two major isoforms, GABAB1a and b, their expressions were expected to be strong. Because GB1 probe sets were designed at the exon junction, there is no possibility of false signals due to genomic contamination. Among commercial primer and probe sets, GB1 sets also best represented the microarray probes that contain exon 23. GB2 sets were specific for other microarray probes containing intron 4. The probes were human cDNA microarray clone 300899 and its mouse homologs. GB3 sets were designed within intron 4 and exon 6. The two primer sets, GB2 and GB3, were used to measure mRNA levels of multiple overlapping splice variants (e.g. primer set GB2 could detect expression of variants GABAB1j, k, l, m, and n while primer set GB3 could detect GABAB1k, l, m, and n except GABAB1j). Therefore, GB2 expression could include GB3.

Quantitative real-time PCR experiments showed different expression patterns between human and mouse brain. In both species, GB2 and GB3 expression was less than GB1 (Figure 4B, C). After comparing GB2 and GB3 expression with GB1, the relative GB2 and GB3 expression in human was less than in mouse (Figure 4B, C). The relative GB3 expression level difference between human and mouse was 4.2 fold which was less than the relative GABAB1j expression difference (25 fold) as shown in Figure 2B.

Prefrontal cortex, frontal cortex, and motor cortex were also used to characterize GB3 expression in human; GB3 expression levels were not significantly different across the brain regions (Figure 4D).

### GABAB1k, GABAB1l, and GABAB1m function

The GABAB receptor is a heterodimeric (GABAB1 + GABAB2) G protein coupled receptor. GABA binding to GABAB1 transfers a signal through G proteins whose functions are linked to several effectors, including GIRK. Thus, GIRK currents were measured using two-electrode voltage clamp to evaluate the functions of the new GABAB1 isoforms.

**Figure 5:**
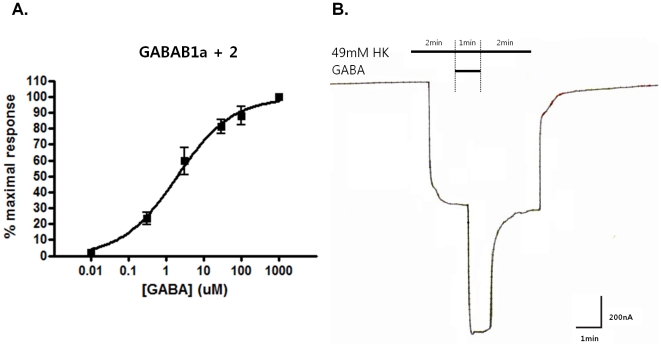
GABAB1a/2 concentration response curve. A. cDNA and cRNA double injections were performed. For cDNAs, both GABAB1a and GABAB2 were injected into the nucleus of *Xenopus* oocytes. GIRK1 and GIRK2 cRNAs were also injected into the cytosol of the same oocytes. Current response of GABAB receptor and GIRK expressing oocytes were measured by two-electrode voltage clamp. 1 mM GABA was the maximum response concentration; EC_50_ was 1.9 uM; Hill slope was 0.59. B. For GABAB1a/2 concentration response curve measurement, GABA dissolved in 49 mM HK was applied for 1 min following a 2 min 49 mM HK application. After 2 additional min of 49 mM HK application, a 20 min wash with ND96 was followed.

GABAB receptor responses were first measured in oocytes expressing GABAB1a and GABAB2 along with GIRK1 and GIRK2 with a range of GABA concentrations. 1 mM GABA was the maximum response concentration. The EC_50_ was 1.9 uM, and Hill slope was 0.59 (Figure 5A).

For functional characterization, GABAB1k, l, and m were chosen because they have more functional domains than GABAB1n. Various amounts of each isoform cDNA were injected into oocytes together with GABAB2, GIRK1, and GIRK2. GIRK currents were measured following the previous GABAB1a and GABAB2 response measurement protocol (Figure 5B). However, no oocyte showed GABA-evoked GIRK currents.

**Figure 6:**
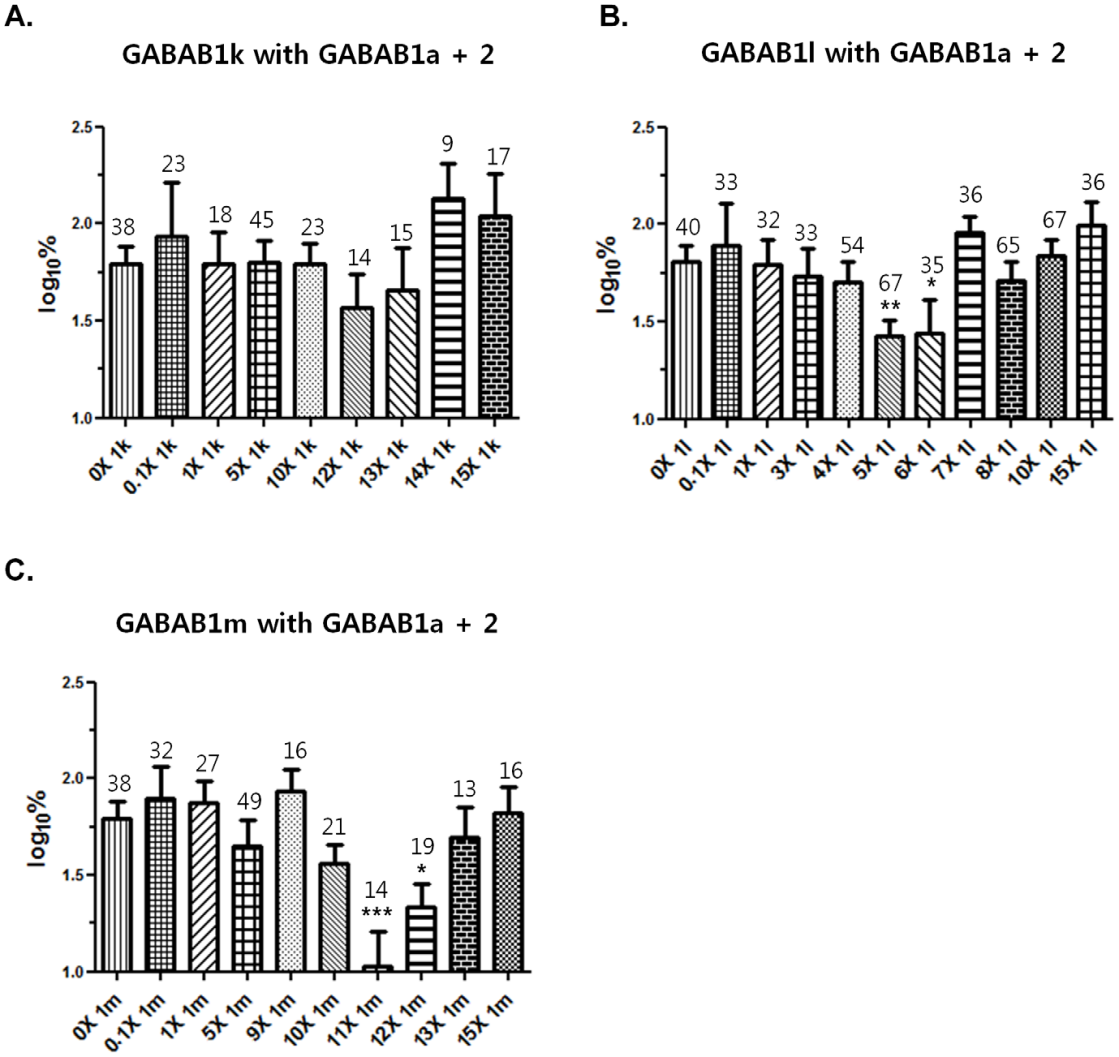
The functional effects of three novel human isoforms, GABAB1k, l, and m. Inhibitory effects were measured in *Xenopus* oocytes 5 days after injecting GABAB1a, GABAB2 cDNA and GIRK1, GIRK2 cRNA along with GABAB1k, l, or m cDNA. For vector controls, vectors were injected in the same amounts as used for the isoforms. Isoform and vector injection amounts varied from 0 to 15 times of GABAB1a injection amount. Used GABA concentration was 3 mM, an approximate maximum response concentration. For each batch, individual GIRK currents from isoform injected oocytes were normalized to percentage by the average vector control current. For nonparametric tests, the individual percent values were changed to log_10_. After one-way ANOVA and Dunnett's tests, one-tailed unpaired Student's t-tests were performed between no isoform injected groups and the isoform injected groups. A. GABAB1k had no inhibitory effect. B. GABAB1l data showed significant differences by ANOVA and Dunnett's tests (p = 0.0058), and caused significantly decreased GIRK currents at 5× and 6× GABAB1l (**p = 0.0014 and *p = 0.0229, respectively). C. ANOVA and Dunnett's tests showed the significant differences of GABAB1m data (p = 0.0040). GABAB1m showed significant decreases at 11× and 12× GABAB1m (***p = 0.0003 and **p = 0.0064, respectively). The numbers of recorded oocytes are shown above the bars by each group. Data are expressed as the mean ± SEM.

Although GABAB1k, l, and m combined with GABAB2 did not induce GIRK currents, we asked if they might inhibit GABAB receptor function. Each isoform cDNA was injected along with GABAB1a, GABAB2, GIRK1, and GIRK2 into oocytes. For vector controls, the same amount of vector (instead of the new isoform) was injected along with GABAB1a, GABAB2, GIRK1, and GIRK2 into the control oocytes. The new isoform- and vector-injected oocytes were paired into groups based on injection amount. Injection amounts varied from 0 to 15 times the amount of GABAB1a injection (Figure 6). After 5 days of incubation, GABA-evoked GIRK currents were measured. GABA, 3 mM - an approximate EC_100_ for GABAB1a and GABAB2 receptors, was used. For each batch of oocytes, individual GIRK currents from the isoform-expressing group were normalized as a percentage of the average current of the vector control group. For nonparametric test, log_10_ values were calculated for the individual percentage values. One-way ANOVA and Dunnett's tests showed significant effects of GABAB1l and m (p = 0.006 and p = 0.004, respectively); however, GABAB1k did not show significant effects (Figure 6A, B, C). For GABAB1l and m data, one-tailed unpaired Student's t-tests were performed between no isoform injection data (0 ×) and the other individual data (0.1× ∼ 15×). GABAB1l significantly decreased GIRK currents at 5× and 6× of the GABAB1a concentration (**p = 0.0014 and *p = 0.023, respectively; Figure 6B). GABAB1m also evoked significant decreases at 11× and 12× of the GABAB1a concentration (***p = 0.0003 and **p = 0.0064, respectively; Figure 6C). Among 9 to 12 different oocyte batches, the inhibitory patterns were consistently observed. Except for the GABAB1l and m injection ranges that decreased GIRK currents, other injection amounts did not show the inhibitory effects. This pattern was also observed at additional injection amounts (8× of GABAB1k, 4.5× and 5.5× of GABAB1l, and 8× of GABAB1m). In the GABAB1l inhibition range (5× ∼ 6× of the GABAB1a concentration), 5.5× also decreased GIRK current. The other injection amounts did not produce inhibition (data not shown), as expected from the inhibitory range shown in Figure 6.

## Discussion

In this study, novel GABAB1 splicing variants containing the intron 4 region were cloned from human and mouse confirming and extending the previous suggestion that this intron is a frequent alternative splicing spot in the rat [6], [7], [13]. Previous studies cloned GABAB1g, i, and j from rat, all of which have a portion of intron 4 [6], [7], [13]. However, intron 4 containing GABAB1 splicing variants were not previously reported from human or mouse. Our quantitative real-time PCR data implied that intron 4 is also an alternative exon in human and mouse. Therefore, mouse GABAB1j clones were found from the cDNA microarray probe set. Though human GABAB1j was not cloned in this study, its existence was confirmed by quantitative real time PCR. From human and mouse, four more intron 4 containing clones were identified. GABAB1k, l, m, and n were cloned from human, and another GABAB1k was also cloned from mouse. Our study further emphasizes that intron 4 is a frequent splicing spot of the GABAB1 gene in multiple species.

Though expression levels of these novel isoforms in total brain were lower than other abundant isoforms (GABAB1a and b), they may be preferentially expressed in specific cell types just as GABAB1a and GABAB1b show specificity for pre- and postsynaptic localization [14] and are localized in the different cells using their own unique promoters [3], [5]. In cerebellum, GABAB1a is predominantly expressed in granule cells, and GABAB1b is abundant in Purkinje cells [4]. Like GABAB1a and GABAB1b, there are many other precedents for N-terminal truncated splicing variants that have their own promoters and show spatially different expression patterns [15], [16], [17], [18], [19]. The novel isoforms are also N-terminal truncated splicing variants and probably use specific promoters for their expression. After identifying GABAB1 gene putative transcription factor binding sites from the PROMO website, possible promoter regions were found on exon 4 and the 5′ part of intron 4 (data not shown) [20], [21]. Thus, the novel isoforms may be restricted to specific cell types and inhibit GABAB receptor function.

GABAB1l and m might be secreted like GABAB1e because they also lack transmembrane domains and have the same C-terminal truncated patterns as GABAB1e [8]. This phenomenon has been observed for splicing variants of Interleukin-15 receptor α, Fibroblast growth factor receptor 4, human GABAB1e, and rat GABAB1j which lose transmembrane domains by alternative splicing and are secreted [7], [8], [22], [23]. Our functional studies used continuous perfusion of the oocytes, and it is possible that GABAB1l and m were secreted and washed away resulting in low intracellular and extracellular concentrations and obscuring some functional effects. However, many secreted splicing variants including the C-terminal truncated GABAB1 isoforms inhibit the functions of their other membrane-bound isoforms [7], [8], [22], [23], [24]. It is possible that secreted GABAB1l, m, and n could also inhibit GABAB receptor function.

The inhibitory effects of GABAB1l and m were found only within a narrow concentration window of injected cDNA. The reasons are not clear, but it is not likely due to nonspecific artifact or toxicity with the highest amounts injected. Therefore, above the optimal concentration window, their effects were diminished. The vector controls also had the same injection amounts, but their GABA responses were constant and stable. Also, the inhibitory effects were seen consistently among all 9–12 different oocyte batches. Some genes require optimal expression levels to display their functional properties [25], [26], [27], and GABAB1m and n may also need optimal expression ranges to inhibit function.

GABAB1e binds GABAB2 though a dimerization motif (166–439 aa of GABAB1e) and appears to dominantly bind GABAB2 as well as competing with GABAB1a [8]. Its dimerization motif is not sushi domains but the rest of the N-terminal extracellular domain. GABAB1k, l, m, n, but not GABAB1j contain the dimerization motif and would be expected to bind GABAB2 dominantly instead of GABAB1a and even reduce receptor function.

In summary, alternative splicings at intron 4 generate novel isoforms, GABAB1j, k, l, m, and n (Figure 1A). The relative mRNA expression patterns of GABAB1k, l, m, and n are more conserved than GABAB1j across species when compared to general GABAB1 isoform expression. However, the expression of novel N-terminal splicing variants, GABAB1k, l, m, and n, is low in whole brain (Figure 4D). They may have restricted expression at specific cell types because they have putative promoter sites. Though GABAB1k, l, m, and n are expected to dominantly dimerize with GABAB2 like GABAB1e, only GABAB1l and m were observed to disrupt the function of GABAB1a and 2. GABAB1e has only C-terminal truncation [8], but the GABAB1l and m have both C-terminal and N-terminal truncated patterns (Figure 1B). Therefore, this additional N-terminal truncated pattern may increase their inhibitory functions.

This work expands evidence for the diversity of GABAB1 splice variants in brain. It is important to note that variants were found in multiple species, and it has been noted that splice variants which are likely to contribute to evolution and have functional value are conserved across species [28]. In considering possible functional roles of these splice variants, we should note that GABAB receptors provide diverse neuronal signaling through G proteins and may have additional functions as they form complexes with other proteins [29].

## Materials and Methods

### Sequence analysis

To predict two possible isoforms, GABAB1j and GABAB1k, SOURCE (http://smd.stanford.edu/cgi-bin/source/sourceSearch) was used to search the sequence of the microarray probe, clone 300899. The sequence was converted to a FASTA format by EditSeq programs of DNASTAR (DNASTAR, Inc., Madison, WI, USA). Blast N Search (www.ncbi.nlm.nih.gov/blast) showed where the probe aligned in GABAB1 gene. Its multiple stop codons were founded from six different translated frames using MapDraw programs of DNASTAR.

To compare GABAB1 isoform sequences, their sequences and exon-intron structure information were found from NCBI data base (http://www.ncbi.nlm.nih.gov). Their sequences were aligned with MegAlign programs of DNASTAR, and their conserved domains were searched by ExPASy Proteomics Server (http://expasy.org) and CD-Search (http://www.ncbi.nlm.nih.gov/Structure/cdd/wrpsb.cgi). To align GABAB1j sequences, ClustalW2 (http://www.ebi.ac.uk/Tools/clustalw2/index.html) were used.

### Identification of new GABAB1 isoform, GABAB1k

To determine the existence of novel GABAB1 isoforms, GABAB1j and GABAB1k, across species, the microarray probe, clone 300899, was cloned from human, and its homologous regions were also cloned from mouse and rat based on Blast N Search. For further examination, partial GABAB1k clones were identified. The partial GABAB1k clones contain the microarray probe and GABAB1k unique region, 3′ intron 4 of GABAB1a. For these experiments, total RNA was isolated from human cultured cells, including HEK-293 (ATCC, Manassas, VA, USA), SH-SY5Y (ATCC, Manassas, VA, USA), and SK-N-SH (ACTT, Manassas, VA, USA). Total RNA was also isolated from mouse midbrain and rat hippocampus. After treating DNase (Qiagen, Valencia, CA, USA), cDNAs were made using oligo(dT) primer (Invitrogen, Carlsbad, CA, USA). To clone the microarray probe, primers were designed within the probe region using Primer3 (http://frodo.wi.mit.edu/primer3/) and PrimerSelect program of DNASTAR. For partial GABAB1k cloning, the probe region and other 3′ exons were be targeted to design primers. PCRs were performed using the primers and the prepared human, mouse, and rat cDNAs.

PCR primers (5′ to 3′):

- Human

Not1-hGABBR1iso-probe-S: AAGGAAAAAAGCGGCCGCCCTTCTTCAGGTTTAAATACTCCA

Sal1-hGABBR1iso-probe-A: ACGCGTCGACCCATCGAGCCCTCTATATTATTAG

Not1-hGABBR1iso-#1probe-S1: AAGGAAAAAAGCGGCCGCCTTGGGCTTTACTTTCCTCACAT

Sal1-hGABBR1iso-#2probe-A1: ACGCGTCGACGAATCTACCAGGGTAGAGGAAGT

Sal1-hGABBR1-A: ACGCGTCGACCTTGATAGGGTCGTTGTAGAGC

Sal1-hGABBR1-A1: ACGCGTCGACAGAGTCGAAGTGAAGACCTCAG

- Mouse

Cla1-mGABBR1iso-probe-S: CCATCGATTAGACTTAGAACATCCTCTGTATCA

Cla1-mGABBR1iso-probe-A: CCATCGATAGAGTTTGGGTGCCCAGAAAAG

Cla1-mGABBR1-A: CCATCGATGGATAAGCTTGAGCTCGTAGTC

- Rat

Not1-rGABBR1iso-probe-S: AAGGAAAAAAGCGGCCGCCCCTCTCTGTAGATGTAGAACATT

Sal1-rGABBR1iso-probe-A: ACGCGTCGACTTCTGGTCATTCTGCCCCAACT

Sal1-rGABBR1-A: ACGCGTCGACGGATAAGCTTGAGCTCGTAGTC

### Cloning novel isoforms, GABAB1k, GABAB1l, GABAB1m, and GABAB1n

To clone full ORFs of the novel isoforms, cDNAs were generated with oligo(dT) primer from human brain total RNA (Ambion, Austin, TX, USA) and mouse midbrain total RNA. Primers were designed based on intron 4 of GABAB1a and common 3′ UTR of known isoforms, and PCRs were performed using the human and mouse brain cDNAs.

PCR primers (5′ to 3′):

- Human

Not1-hGABBR1iso-#1probe-S1: AAGGAAAAAAGCGGCCGCCTTGGGCTTTACTTTCCTCACAT

Sal1-hGABBR1-A2: ACGCGTCGACGGTAACTAGAAGAGGGTGTTGC

Not1-hGABBR1cl-CD-S: AAGGAAAAAAGCGGCCGCTCTTCTCTGATCCCCGTCTTT

Sal1-hGABBR1cl-CD-A: ACGCGTCGACTACTGGCCTGTCCTCCCTC

- Mouse

Cla1-mGABBR1cl-CD-S: CCATCGATTTCTCTGATCCCCGTCTTTC

Cla1-mGABBR1cl-CD-A: CCATCGATTGCTGGCCTCATCCTTCTC

### Quantitative real-time PCR

TaqMan® Gene Expression Assays and Custom TaqMan® Gene Expression Assays (Applied Biosystems, Foster City, CA, USA) were used to detect GABAB1 isoform expressions. cDNA microarray probes are two different kinds containing exon 23 or intron 4. The probes containing exon 23 detect all known isoform expressions except GABAB1j. The exon 22–23 junction was targeted using predesigned TaqMan® Gene Expression Assays. Custom TaqMan® Gene Expression Assays were used for intron 4 containing probes, and thus were able to detect GABAB1j, GABAB1k, GABAB1m, GABAB1l, and GABAB1n. Using the same method, the unique alternative splicing region of GABAB1k, GABAB1m, GABAB1l, and GABAB1n were targeted, and GABAB1j specific expression was also detected. For endogenous control gene, beta-glucuronidase (*GUSB*) was used.

After making cDNAs with random primers (Invitrogen, Carlsbad, CA, USA), quantitative real-time PCR was performed. Isoform expressions were compared between human and mouse whole brain RNAs (Ambion, Austin, TX, USA). To measure isoform expression differences in select human brain regions, RNAs from whole brain, prefrontal cortex, frontal cortex (Ambion, Austin, TX, USA), and motor cortex [30] were used.

### cDNA and cRNA preparation

To do injections into *Xenopus* oocytes for electrophysiological recordings, cDNAs and cRNAs were prepared. For cDNA injection, human GABAB1a clone in pcDNA3.1(−) vector (Invitrogen, Carlsbad, CA, USA) and human GABAB2 clone in pcDNA3 vector (Invitrogen, Carlsbad, CA, USA) were kindly provided by Dr. Uezono (Nagasaki University, Nagasaki, Japan) with the permission of GlaxoSmithKline (Brentford, Middlesex, UK). The cDNAs encoding novel human GABAB1 isoforms, GABAB1k, GABAB1l, and GABAB1m, were also subcloned into pcDNA3.1(−) vector. The cRNAs for GIRK1 and GIRK2 were synthesized as described previously for cRNA injections [31].

### Electrophysiological recording

For electrophysiological recordings, oocytes from *Xenopus laevis* frog were used. The frog was used according to the National Institutes of Health guide for the care and use of laboratory animals.

To form functional receptors, nuclear injection of GABAB1a and GABAB2 cDNAs were performed into *Xenopus* oocytes with 0.75 ng and 0.63 ng, respectively. GIRK1 (0.2 ng) and GIRK2 (0.2 ng) cRNAs were also injected into the cytosol of the same oocytes in order to measure GABAB receptor-induced currents. Total injection volumes of cDNAs and cRNAs were 60 nl. Electrophysiological recordings were made with the oocytes after 5 to 7 day incubation at 19°C. Using two-electrode voltage clamp, the oocytes were clamped at −60 mV while being perfused (2 ml/min) with ND96 buffer (in mM: 96 NaCl, 2 KCl, 1 CaCl_2_, 1 MgCl_2_, 5 HEPES, pH7.5). A 49 mM high-K^+^ solution (49mM HK) (in mM: 48 NaCl, 49 KCl, 1 CaCl_2_, 1 MgCl_2_, 5 HEPES, pH7.5) was applied to the oocytes for 2 min [32]. GABA (Sigma, St. Louis, MO, USA), ranging in concentration from 10 nM to 1 mM, was dissolved in 49 mM HK and applied for 1 min. ND96 was applied for 20 min following the 2 min application of 49 mM HK.

The responses of GABAB1a and GABAB2 to the range of GABA concentrations were used to generate GABA concentration response curves with GraphPad Prism, Version 5.01 (GraphPad Software, San Diego, CA, USA). EC_50_ and Hill coefficient values were calculated from the GABA concentration response curves. A maximally effective GABA concentration was also determined from it and was used to test inhibitory effects of the isoforms.

To test if the isoforms can substitute for GABAB1a as a functional receptor subunit, 0.63 ng GABAB2, 0.2 ng GIRK1, and 0.2 ng GIRK2 were injected into oocytes with various amounts (0.001 ng to 20 ng) of isoform cDNAs. Total injection volumes were 60 nl. As in the GABA concentration response experiment, GABA-evoked GIRK currents were measured to determine receptor function.

To determine the inhibitory functions of isoforms, GABAB1a was co-injected with each isoform. Except the isoforms, the injection amounts of GABAB1a, GABAB2, GIRK1, and GIRK2 were the same as the GABA concentration response experiment. For vector controls, the same amount of vector, pcDNA3.1(−), was injected into control oocytes instead of the isoforms. Total injection volumes were 60 nl. The isoform and vector injection amounts varied from 0 to 15 times the GABAB1a injection amount (0.75 ng). After a 5 day incubation at 19°C, GABA-evoked GIRK currents were measured. The GABA concentration was 3 mM which was the maximal concentration for GABA responses in GABAB1a and GABAB2 expressing oocytes. Nine batches of oocyte were used for both GABAB1k and m experiments, and 12 batches were for GABAB1l. For each batch of oocytes, individual GIRK currents in isoform injected oocytes were expressed as a percentage of average current in vector control oocytes. For nonparametric tests, log_10_ data were calculated from the values.

### Statistical analysis

For statistical analysis and graphing, GraphPad Prism and Excel (Microsoft, Redmond, WA, USA) were used. For electrophysiology data, one-way ANOVA tests and unpaired Student's t-tests were used to define statistical significances. Dunnett's tests were also used for post hoc tests.
